# Differential immunohistochemical expression of type I collagen and matrix metalloproteinase 2 among major salivary glands of young and geriatric mice

**DOI:** 10.1590/1678-7757-2017-0484

**Published:** 2018-05-07

**Authors:** Mehmet Kemal TUMER, Mustafa CICEK

**Affiliations:** 1 Gaziomanspasa University, Faculty of Dentistry, Department of Oral and Maxillofacial Surgery, Tokat, Turkey.; 2 Gaziosmanpasa University, Faculty of Medicine, Department of Medical Biology, Tokat, Turkey.; 3 Kahramanmaras Sütcü Imam University, Faculty of Medicine, Department of Anatomy, Kahramanmaras, Turkey.

**Keywords:** Parotid gland, Type 1 collagen, Salivary glands, Matrix metalloproteinase 2, Aging

## Abstract

**Objective:**

This study aimed to demonstrate the immunohistochemical changes associated with MMP-2 and type 1 collagen separately for the first time in the major salivary glands (the parotid, submaxillary, and sublingual glands) that occur with aging in mice.

**Material and Methods:**

Fourteen Balb/c white mice (50-80 g) were used in this study. The animals were divided into two equal groups. Group I consisted of young animals (2-month-old) (n=7) and Group II consisted of older animals (18-month-old) (n=7). After routine histological follow-ups, Hematoxylin-eosin (H&E), Masson’s Trichrome staining and immunohistochemical staining was performed for type I collagen and MMP-2.

**Results:**

We observed that there were age-related decreases in the number of acinar cells, increase in eosinophilic zymogen granules in cells, collagen accumulation in fibrotic areas and dilatation in interlobular ducts. Also, while type I collagen and MMP-2 immunoreactivity were moderate in the salivary glands of the young mice, they were high in the salivary glands of the old mice (p=0.001). In the H-score assessment, MMP-2 immunoreactivity was lower at a significant level in young mice than in old mice (p=0.001).

**Conclusions:**

This study showed that anatomical, physiological and morphological abnormalities occur in all three major salivary glands as a natural consequence of aging.

## Introduction

Three pairs of major salivary glands produce saliva (the parotid, submandibular, and sublingual glands) as well as minor salivary glands located throughout the oral cavity^1^. The protective characteristics against bacteria, viruses and fungi depend on the effect of salivary antibodies (mostly secretory IgA) and enzymes (lysozyme, lactoferrin, histatin, etc.) as well as adhesive properties of mucin^2^ ^-^ ^5^. The major salivary glands are branched tubuloacinar glands. They are composed of acini (secretory part) and channel systems (transmitter part). In acini with tubular (mucous acinus) and spherical (serous acinus) structure, there are myoepithelial cells (non-secretory) and serous or mucous cells (secretory) or both types of cells. The main secretory units of salivary glands consist of serous and mucous cells. Salivary secretion can be classified as serous, mucous, or mixed^6^. Although the sublingual gland is a mixed gland, the mucous cells are predominant, and therefore it is rich in mucin secretion^7^.

Matrix metalloproteinases (MMPs) play an important role in physiological conditions such as tissue remodeling, morphogenesis, wound healing and normal developmental processes, as well as pathological processes such as tumor cell invasion, angiogenesis and metastasis^8^. Some publications have shown that MMP-2 (Gelatinase A), which is found at a molecular weight of 72 kDa in its pro-form and 66 kDa in the active form, degrades gelatin and type IV collagen as well as type I, II and III collagen^9^.

Collagens are the principal proteins of the extracellular matrix, which constitute 30% of the dry body weight. They provide a great balance of flexibility and strength to the tissue in which they are found. Type I collagen is the member of the collagen family, which is the most abundant and widely distributed in fibril form^10^.

There are many publications examining the changes in the structure and function of organs during aging. In publications about human and animal salivary glands, only a single gland is usually emphasized when mentioning the changes of salivary glands with aging. However, specifying differences between the major and minor glands as well as evaluating the major salivary glands individually among themselves increases the accuracy of structural and functional studies to be performed on salivary glands. Since these glands, the functions of their secretions and the quantities are different from each other, rates and contents of their secretions also vary according to the gland. This indicates that they must be assessed separately rather than together as in previous studies. This study aimed to demonstrate the immunohistochemical changes associated with MMP-2 nd type 1 collagen separately for the first time in the major salivary glands (the parotid, submaxillary, and sublingual glands) with aging in mice.

## Material and methods

### Supply of animals

The study was approved by the institutional Animal Care and Ethics Committee of Gaziosmanpasa Medical University (HADYEK- 34) and was conducted in the experimental research unit. The animal study strictly adhered to the animal experiment guidelines of the Committee for Animal Care. Fourteen Balb/c white mice (50-80 g) were used in this study. All of them were female mice. The animals were divided into two equal groups of seven animals. Group I consisted of young animals (2-month-old) (n=7) and Group II consisted of old animals (18-month-old) (n=7). The animals were kept at room temperature (22±1°C) and 40-50% humidity before the application. Light level was set to cycle between 12-h light and 12-h dark. The animals were set free to eat and drink. The mice were kept under observation for 1 week and daily physical examination was performed. All the animals were decapitated approximately at 01:00 pm to eliminate any circadian rhythm related effects.

### Obtaining samples

The mice without any health problems were killed by cervical dislocation technique under anesthesia induced with ketamine/xylazine (50/10 mg/kg). The major salivary glands were separately removed. They were taken in 10% formalin for use in immunohistochemical and histopathological evaluations.

### Histological examination of salivary gland tissues

The salivary gland tissues were removed and were taken in 10% formalin. After routine histological follow-ups, tissues were embedded in paraffin. The 4-5-μm-thick tissue sections were taken from the paraffin-embedded tissues. Then, Hematoxylin-eosin (H&E) and Masson’s Trichrome staining were applied to the sections. The stained sections were examined with the Carl Zeiss Axio Imager A1 light microscope (Carl Zeiss Ltd., Cambridge, MA, USA).

### Immunohistochemical examination

The 4-5-mm-thick sections taken from paraffin blocks were transferred onto poly-lysinized lamellae. After the deparaffinized tissues were dehydrated by passing them through a series of increasing alcohol concentrations, the tissues rinsed in distilled water were boiled in citrate buffer solution (pH:6.0) for 5 minutes in a microwave oven (600 W) for antigen retrieval. They were treated with H_2_O_2_ to block endogenous peroxidase activity. To prevent background staining, they were treated with Ultra V Block (Ultra V Block, TA-125-UB, Thermo Fisher Scientific Inc, USA) solution, and then were incubated with primary antibody (Rabbit polyclonal to Tip I Collagen, Abcam, ab34710, Camridge, UK; Rabbit polyclonal to MMP-2, Abcam, ab37150, UK) for 60 minutes. After primary antibody application, secondary antibody (biotinylated anti-mouse IgG, Diagnostic BioSystems, KP 50A, Pleasanton, USA) and streptavidin horseradish peroxidase were applied for 30 minutes. After 3-amino–9-ethyl carbazole chromogen was applied, contrast staining was carried out using Mayer’s hematoxylin. Instead of primary antibody, phosphate-buffered saline (PBS) was used in tissues prepared for negative control. The other steps were applied in the same way. The tissues treated with PBS and distilled water were covered with an appropriate covering solution. The prepared slides were examined with Carl Zeiss Axio Imager A1 light microscope (Carl Zeiss Ltd., Cambridge, MA, USA); they were also evaluated and photographed.

We performed imunohistochemical assay using the H-score analysis^11^. The intensity of MMP-2 and type 1 collagen immunoreactivity was evaluated semi-quantitatively by using the categories of stain intensity during follow-up: 0 (No staining), 1+ (Weak but detectable staining), 2+ (Moderate staining), and 3+ (Intensive staining). Firstly, an H-score value was obtained for each tissue by calculating the sum of the percentages of cells according to the categories of stain intensity. Then, this value was multiplied by the weighted intensity of staining using the H-score formula =ΣPi (i 1+) (“i” represents the intensity scores and “Pi” represents the relative percentage of cells). Each slide was evaluated in 5 randomly selected areas in light microscope (40X magnification). The percentage of cells in each intensity in these areas was identified at different times by two researchers who were unaware of the species and source of the tissues. The average scores of both researchers were used.

### Statistical analysis

The Shapiro-Wilk test was used for testing normality. The data were normally distributed. P values <0.05 were considered statistically significant. The independent two-sample t-test was used to compare groups.

## Results

### Histological findings

#### Hematoxylin-eosin staining

**Figure 1:**
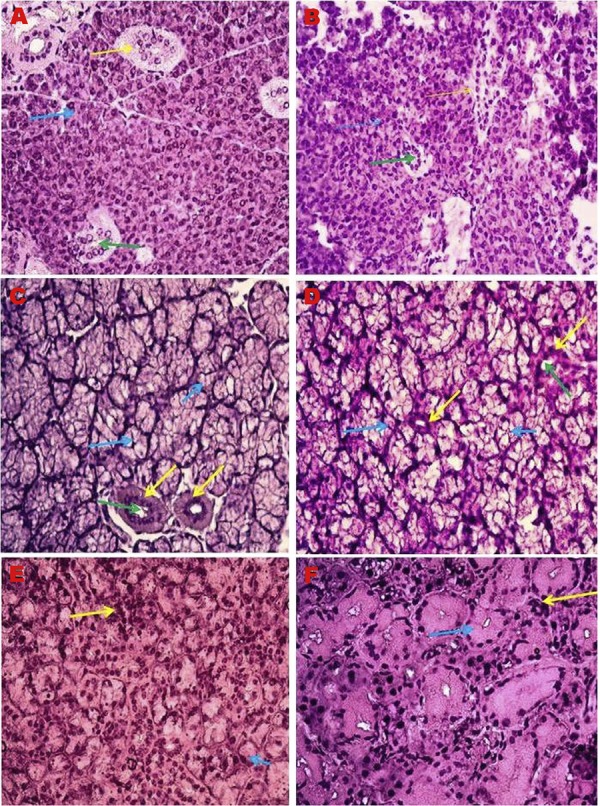
Major salivary gland histopathology H&E, 40X. A) Parotid gland of young mice. *Yellow arrow: Straited excretory duct; Blue arrow: Serous acini; Green arrow: Striated duct lumen; B) Parotid gland of old mice. * Yellow arrow: Straited excretory duct; Blue arrow: Serous acini; Green arrow: Striated duct lumen; C) Sublingual gland of young mice. * Yellow arrow: Straited excretory duct; Blue arrow: Mucous acini; Green arrow: Striated duct lumen; D) Sublingual gland of old mice. * Yellow arrow: Straited excretory duct; Blue arrow: Mucous acini; Green arrow: Striated duct lumen; E) Submandibular gland of young mice. *Yellow arrow: Serous acini; Blue arrow: Mucous acini; F) Submandibular gland of old mice; *Yellow arrow: Serous acini; Blue arrow: Mucous acini

Histological structure of the major salivary glands was photographed by a light microscope in young and old animals (Figure 1). We observed in the submandibular and sublingual glands of young mice that the pale bubble-like cytoplasms were present and the nuclei were pushed towards the basement membrane. The cytoplasms of serous cells contained prominent eosinophilic zymogen granules and the nuclei had a spherical appearance. Swollen mucosal cells and serous cells surrounding them in a demilune position were observed. There was a visible decrease in the number of acinar cells in the submandibular and sublingual glands of old mice. An increase in the volume of granular convoluted tubules due to aging was observed. The shrunken nuclei, enlarged cytoplasms, disrupted cell boundaries and smaller cell areas were detected in all cell types in old mice. Moreover, blood vessels decreased and shrank due to aging. Increased fat tissue in the parotid glands of the old mice was observed. In the parotid gland, we also observed that the parenchymal tissue was divided into many lobules by the stromal connective tissue. Blood vessels and large interlobular ducts were seen in the stromal connective tissue. Interlobular channels were lined with pseudostratified epithelium.

#### Masson’s trichrome staining

**Figure 2:**
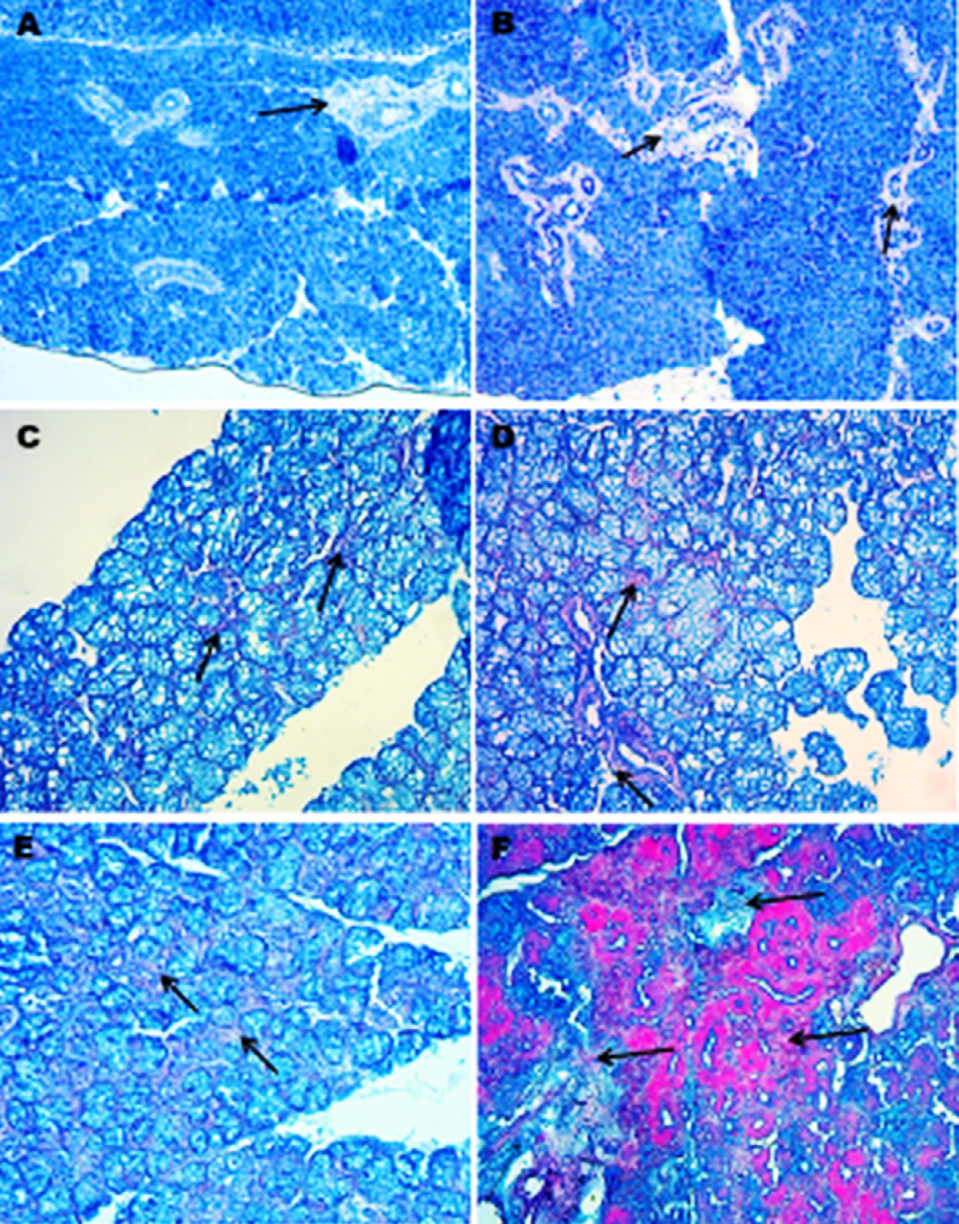
Major salivary gland Masson’s trichrome staining, 20X. A) Parotid gland of young mice; B) Parotid gland of old mice; C) Sublingual gland of young mice; D) Sublingual gland of old mice; E) Submandibular gland of young mice; F) Submandibular gland of old mice; *Black arrow: Collagen fibers

To assess type I collagen, Masson’s trichrome staining was used^12^. Fibrotic areas are rich in collagens and, therefore, they appear in blue upon Masson trichrome staining (asterisks). We observed that the total collagen density was lower in young mice than in older mice. The collagen fibers were looser and disorganized in older mice than in young mice. Moreover, acinar atrophy and dilated interlobular ducts were seen in old mice (Figure 2).

## Immunohistochemical findings

**Figure 3:**
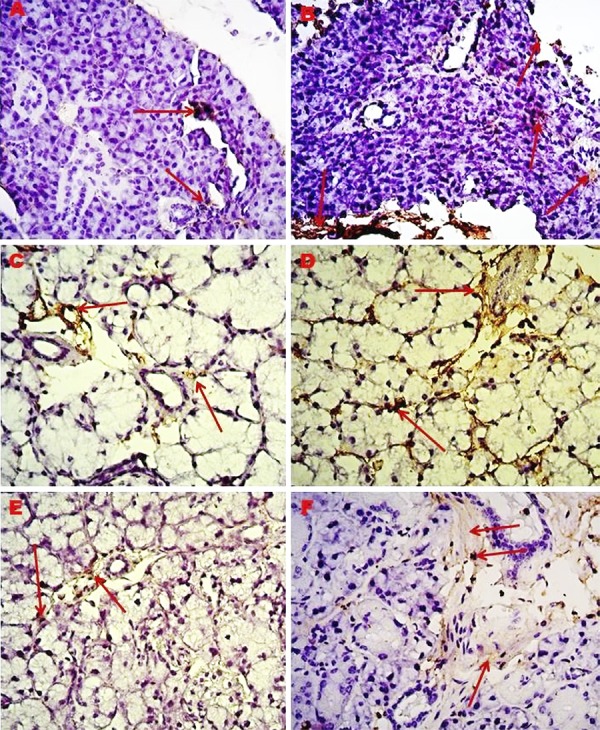
Immunreactivity of type I collagen protein in major salivary gland, 40X. A) Parotid gland of young mice; B) Parotid gland of old mice; C) Sublingual gland of young mice; D) Sublingual gland of old mice; E) Submandibular gland of young mice; F) Submandibular gland of old mice; *Red arrow: Collagen fibers

Immunohistochemical staining for type I collagen and MMP-2 was performed in salivary gland tissue sections of young and old mice. No immunoreactivity was observed in the negative control staining. The results were evaluated semiquantitatively and are shown in Table 2. While the salivary gland tissues of old mice showed a high immunoreactivity (+++) for type I collagen and MMP-2, they showed a moderate immunoreactivity (++) in young mice (Figures 3 [Fig f04] to 5). In the H-scoring results performed by immunopositive staining of tissue sections of the parotid, submandibular, and sublingual glands forming the salivary glands, MMP-2 immunoreactivity was lower at a significant level in young mice than in older mice (p=0.001) (Table 1, Figure 6).

**Table 2:** Comparison of immunreactivity of young and old mice

		Group				Total		
		Young (n:7)		Old (n:7)		(n:14)		
intensity		n	%	n	%	n	%	P
Major Salivary Gland Sublingual Type 1 Collagen	+	7	100	0	0	7	100	0.001*
	+++	0	0	7	100	7	100	
Major Salivary Gland Sublingual MMP 2	+++	0	0	7	100	7	100	0.001*
	+	7	100	0	0	7	100	
Major Salivary Gland Submandibular Type 1 Collagen	+	7	100	0	0	7	100	0.001*
	+++	0	0	7	100	7	100	
Major Salivary Gland Submandibular MMP 2	+++	0	0	7	100	7	100	0.001*
	++	7	100	0	0	7	100	
Major Salivary Gland Parotid Type 1 Collagen	+	7	100	0	0	7	100	0.001*
	++	0	0	7	100	7	100	
Major Salivary Gland Parotid MMP 2	+++	0	0	7	100	7	100	0.001*
	+	7	100	0	0	7	100	

*Statistically significant; p<0.05; Fisher Exact test

**Table 1:** Comparison of H-score values of young and old mice

	Young (n:7)	Old (n:7)		
	**x̄ ± SD**	**x̄ ± SD**	**t**	**p**
Submandibular MMP 2	153.14±14.69	171.57±8.98	2.832	0.015*
Sublingual MMP2	120.57±16.55	151.29±18.35	3.289	0.006*
Parotid MMP 2	55.43±12.27	95.14±9.28	6.829	0.001*

*Difference is statisticallly significant; p<0.05: independent samples t test

**Figure 4 f04:**
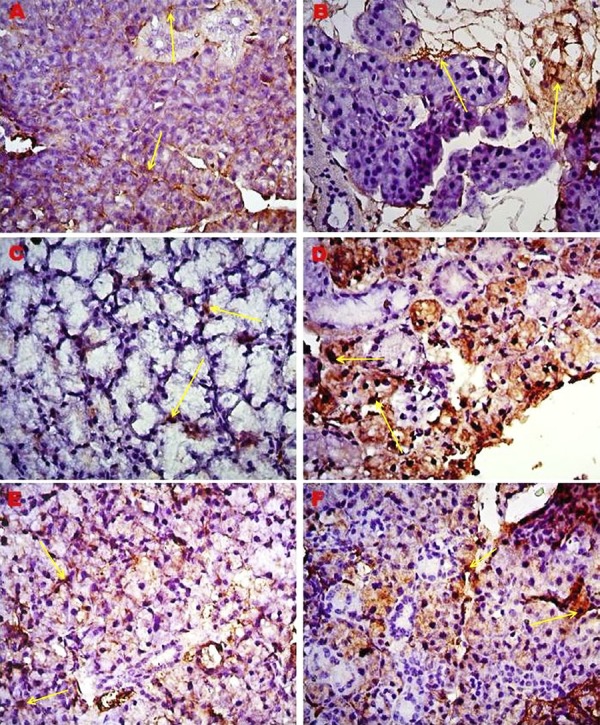
Immunreactivity of MMP-2 protein in major salivary gland, 40X. A) Parotid gland of young mice; B) Parotid gland of old mice; C) Sublingual gland of young mice; D) Sublingual gland of old mice; E) Submandibular gland of young mice; F) Submandibular gland of old mice; *Yellow arrow: Stained cell nuclei

**Figure 5:**
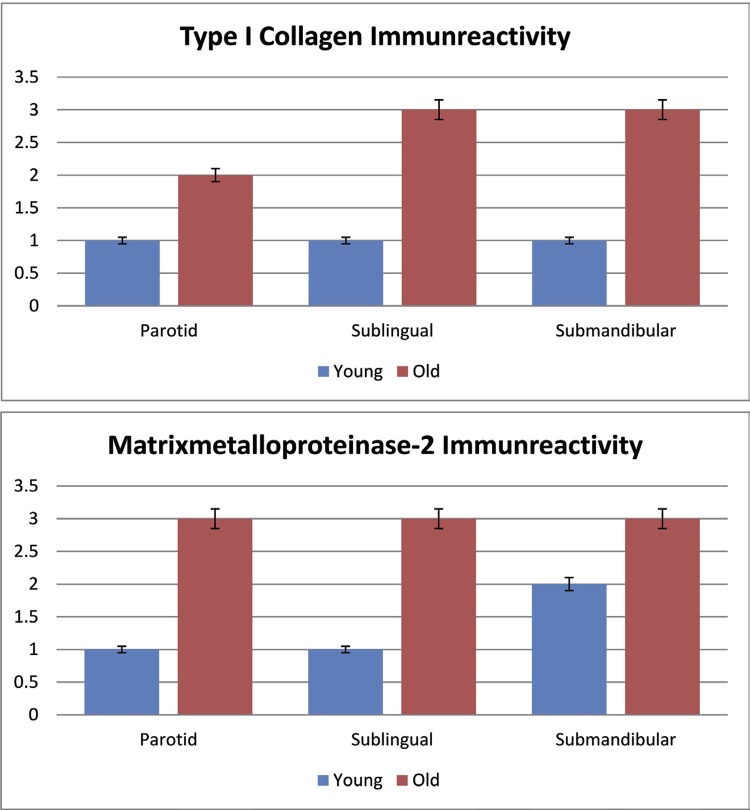
Comparison of immunreactivity of MMP-2 and type I collagen in major salivary gland of young and old mice

**Figure 6:**
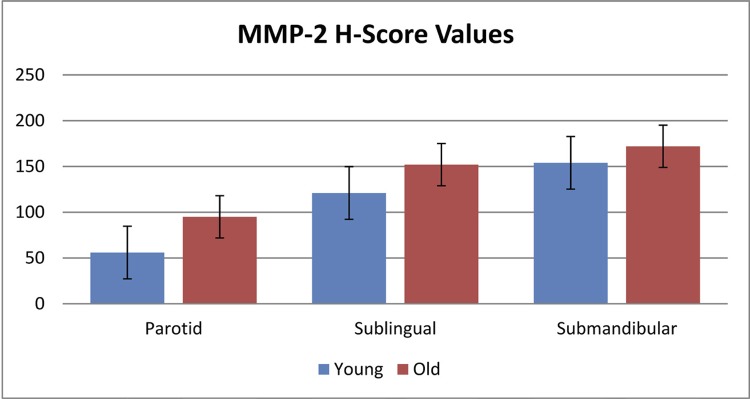
H-score of MMP-2 immunoreactivity in major salivary gland of young and old mice

## Discussion

Salivary secretion can be altered by physiological, psychological, and various environmental stimuli^1^. Aging is one of the main physiological factors for saliva reduction. Among its many function, salivary secretion can protect the integrity of oral tissues^13^. It is very effective in protecting teeth against decay, tasting, swallowing, talking, and adapting to prosthetics^14^. We observed in this study that MMP2 and type 1 collagen expression in the major salivary glands significantly increased in older mice (eighteen-month-old) than in young mice (two-month-old). According to this information, in many studies examining salivary glands in humans and animals, only a single gland is usually emphasized when mentioning the changes of salivary glands with aging. This study is the first study to compare immunohistochemically and separately the MMP2 and Type 1 collagen expression in the major salivary glands of young and old mice with aging.

We performed morphometric evaluations of age-related salivary gland dysfunction using *in vivo* mouse model. Mice were used in translational experimental studies because of their widespread use, their easy availability, and their similarities to humans in terms of anatomy, physiology, and genetics. In the studies conducted, the mean life span of small Balb/c (Albino) mice was shown to be between 18 and 20 months for both sexes under conventional conditions^15^. Many authors have shown that 30 days are equivalent to a day for murine^16^ ^,^ ^17^. In this study, a complete aging model was established using 18-month-old Balb/c mice.

The microscopic changes in salivary glands, which are related with age, were reported in mice. They include the displacement of the parenchymal tissue by acinar atrophy in fat and connective tissues, increased number of ducts, focal and diffuse mononuclear infiltration, and blocked blood vessels. In a study examining the sublingual glands, rare findings such as acinar autolysis and mucus extravasation in addition to age-related conditions have been reported^18^. A study examined the sublingual glands of mice showed signs of temporary acinar autolysis^19^. As in other studies, this study showed that the aging process has negative effects, such as acinar atrophy, lymphocyte infiltration and high-grade periductal fibrosis in acinar and ductal cells in salivary glands. Since this study showed loss of cell boundaries regardless of its degree in the salivary glands of old mice, we observed that acini were autolyzed and mucous extravasation was distributed in the glandular parenchyma. This can be related to acinar arrhythmia. According to our research, there are no studies in which all three major salivary glands were examined histopathologically and separately in mice.

In a separate study, the authors performed Masson’s trichrome and alcian blue staining in only the submaxillary salivary gland and drew attention to decreased amount of mucin and increased periductal fibrosis. In the same study, the authors performed TUNEL staining in old submaxillary gland and mentioned an increase in aging-related apoptosis^20^. However, this study was performed only in the submaxillary gland and this prevents the evaluation of findings about all salivary glands. Because of Masson’s trichrome staining that was performed in this study, fibrotic regions were rich in collagens and it has been investigated that the total collagen density was lower in young mice than in older mice. In addition, acinar atrophy and dilated interlobular ducts were observed in old mice.

In a study conducted on aging, it was reported that hydroxyproline content in the submandibular gland significantly increased on the 14^th^ postnatal day. It was shown that chains of type I collagen were present until the 6^th^ postnatal day. While type III collagen was dispersedly expressed in the interlobular connective tissue from the 7^th^ postnatal day, type IV collagen content increased. The same study reported that during fetal stage, rat submandibular gland types I and IV collagen and laminin were already present and related to the construction of the tissue^21^. In another study, the parotid glands of non-diabetes mellitus (NDM) rats were compared with that of diabetes mellitus (DM) rats. These authors reported that wide intervals between acini, large amounts of collagen accumulation and intensely stained thick type I collagen fibers were seen in the parotid glands of the DM group^22^. In this study, while the salivary gland tissues of old mice showed a high immunoreactivity (+++) for type I collagen, they showed a moderate immunoreactivity (++) in young mice. We observed that collagen fibers were looser and disorganized in older mice than in young mice.

It is known that matrix metalloproteinases (MMPs) cleave components of the extracellular matrix (ECM). Several studies have shown that disruption of epithelial cell-matrix interactions may lead to reduction of cell growth and cell death^23^. Salivary duct cells express large amounts of MMP-2. Unlike the ductal epithelium, acinar cells cannot express MMP-2. This expression model is part of observations of Azuma, et al.^6^ (1997), who describe that ductal cells consistently express MMP-2 and MMP-9, but acinar cells weakly express MMP-2 and MMP-9. For this reason, the ductal epithelium seems to be a source of MMP activity detected in saliva taken from a healthy individual^7^. In this study, the borders of acinar cells increased in old mice due to aging. According to the H-score assessment of this study, MMP-2 (+) cell number and MMP-2 immunoreactivity in the salivary ducts significantly increased in older mice than in young mice.

The extracellular matrix and the basement membrane associated with epithelial cells play a critical role in morphogenesis and in distinguishing developing salivary glands. In early investigations, *ex vivo* organ culture and tissue recombination method were used to determine the importance of ECM in organ development^8^. Recent studies have identified changes related to the extracellular matrix, basement membrane and proteins in salivary gland diseases, including salivary gland carcinomas and Sjögren’s syndrome^24^.

Matrix metalloproteinases and tissue inhibitors of metalloproteinases are required in the fibrosis process depending on their functions in extracellular matrix turnover in various organs. Some studies reported that these components showed efficacy in chronic obstructive sialadenitis of the submandibular gland. When inflammatory salivary gland lesions were compared with a normal salivary gland, we observed that there was an increase in immunoreactivity for matrix metalloproteinases 2, 3, 9, and 13 and tissue inhibitors of metalloproteinases^25^. In parallel with these results, this study observed an increase in MMP-2 immunoreactivity in all salivary glands due to aging. In addition to significant extracellular matrix remodeling in salivary glands in patients with Sjögren’s syndrome, significant changes can be detected in the morphology and function of acinar and ductal cells. Some studies have shown that matrix metalloproteinases influenced the stroma of lip salivary glands, matrix proteins and basal membranes in Sjögren’s syndrome. In this study, we found that there was an increase in proteolytic activity on basal membrane and stromal proteins, most prominent change in fibronectin, laminin and type I collagen^26^. Similarly, type I collagen immunoreactivity was significantly higher in old mice and there was deterioration in acinar and ductal cells with aging.

## Conclusion

Consequently, this study showed that anatomical and morphological abnormalities occurred in the salivary glands with aging because of histopathological and immunoreactive evaluations of each of the three major salivary glands. In addition, reduced salivary gland activity had considerable importance in dysfunctions in oral tissue integrity, dental decay, swallowing, talking, and adapting to prosthetics. In this context, serious pain complications and functional disturbances may occur with losses of physiological activity due to age-related degeneration in the major salivary glands.
